# Structural Characteristics and Cementitious Behavior of Magnesium Slag in Comparison with Granulated Blast Furnace Slag

**DOI:** 10.3390/ma17020360

**Published:** 2024-01-11

**Authors:** Ping Lu, Yueqi Zhao, Na Zhang, Yidi Wang, Jiale Zhang, Yihe Zhang, Xiaoming Liu

**Affiliations:** 1 Engineering Research Center of Ministry of Education for Geological Carbon Storage and Low Carbon Utilization of Resources, China University of Geosciences, Beijing 100083, China; 2 Beijing Key Laboratory of Materials Utilization of Nonmetallic Minerals and Solid Wastes, China University of Geosciences, Beijing 100083, China; 3 National Laboratory of Mineral Materials, China University of Geosciences, Beijing 100083, China; 4 School of Materials Science and Technology, China University of Geosciences, Beijing 100083, China; 5 School of Metallurgical and Ecological Engineering, University of Science and Technology Beijing, Beijing 100083, China

**Keywords:** magnesium slag, granulated blast furnace slag, structural characterization, nuclear magnetic resonance, cementitious activity

## Abstract

Magnesium slag is a type of industrial solid waste produced during the production of magnesium metal. In order to gain a deeper understanding of the structure of magnesium slag, the composition and microstructure of magnesium slag were investigated by using characterization methods such as X-ray fluorescence, particle size analysis, X-ray diffraction, Fourier transform infrared spectroscopy and scanning electron microscopy. In addition, the state of Si occurrence in magnesium slag was analyzed using a solid-state nuclear magnetic resonance technique in comparison with granulated blast furnace slag. An inductively coupled plasma-optical emission spectrometer and scanning electron microscope with energy dispersive X-ray spectroscopy were used to characterize their cementitious behavior. The results show that the chemical composition of magnesium slag mainly includes 54.71% CaO, 28.66% SiO_2_ and 11.82% MgO, and the content of Al_2_O_3_ is much lower than that of granulated blast furnace slag. Compared to granulated blast furnace slag, magnesium slag has a larger relative bridging oxygen number and higher [SiO_4_] polymerization degree. The cementitious activity of magnesium slag is lower compared to that of granulated blast furnace slag, but it can replace part of the cement to obtain higher compressive strength. Maximum compressive strength can be obtained when the amount of magnesium slag replacing cement is 20%, where the 28-day compressive strength can be up to 45.48 MPa. This work provides a relatively comprehensive analysis of the structural characteristics and cementitious behavior of magnesium slag, which is conducive to the promotion of magnesium slag utilization.

## 1. Introduction

Magnesium slag is an industrial solid waste generated during the production of magnesium metal. In China, the smelting of magnesium metal via the Pijiang method is the main commercial process for magnesium production [1,2]. More than 6 tons of magnesium slag is produced when smelting 1 ton of magnesium metal on average, and it is accompanied by nearly 30 tons of CO_2_ and various types of flue gas emissions [3], which seriously pollute the environment of the area where it is located [4]. At present, there is no complete treatment technology for magnesium slag to enable its industrial utilization, which leads to massive accumulation of magnesium slag in the open air [5]. This not only takes up considerable land resources and damages the ecological environment, but also poses a threat to human health [6,7,8,9]. Like other industrial solid wastes, if magnesium slag can be effectively utilized, this will bring great ecological and economic benefits [10,11,12,13,14], promoting the early realization of China’s goal of “carbon peak and carbon neutrality” according to the Paris Agreement.

Magnesium slag mainly contains CaO, SiO_2_ and MgO. Due to its high MgO content, expansion behavior occurs during the hydration process [15,16,17], and for that reason, magnesium slag is not yet used on a large scale in the construction industry. Ji et al. [18] discussed the potential of magnesium slag as a mineral admixture and found that when 30% of magnesium slag was used as a replacement for Portland cement, this was beneficial to improve the late strength of concrete and reduce drying shrinkage. Xie et al. [19] used magnesium slag as an admixture for low-carbon cement, and the prepared samples met the GB 175-2007 [20] “General Portland Cement” standard, which could reduce the production cost by more than 10%. The synthesis of porous materials from magnesium slag can have a good adsorption effect on Pb^2+^ while the materials also have high compressive strength [21]. CO_2_ solidified fiber cement boards prepared with magnesium slag as a binder had high flexural strength, carbonation rate and water absorption [22]. Using the “leaching-carbonization” method, magnesium slag can be turned into two value-added products: vaterite with a purity of more than 95% and supplementary cementitious materials [23]. Magnesium slag has limited activity in its normal state, making it difficult to be applied directly. Therefore, scholars use diverse approaches to stimulate its activity and improve applicability. Lei et al. [24] proposed CO_2_ activated aerated concrete with a high admixture of magnesium slag, which is capable of achieving rapid carbonation to improve compressive strength, reduce environmental pollution caused by the accumulation of magnesium slag and also facilitate the large-scale utilization of CO_2_. When cured in water at 60 °C [25], a magnesium slag product treated with CO_2_ activation has good volume stability and does not display excessive expansion. The effect of volumetric instability can be eliminated after carbonation for 2 h. At the same time, carbonation treatment can quickly obtain higher compressive strength, reaching 90 MPa at 24 h [26]. The incorporation of magnesium slag can improve the soil environment by granularizing the soil and providing higher cementitious activity [27,28,29], and also has a remediation effect on Cd- and As-contaminated paddy soils [30]. Jia et al. [31] investigated the desulfurization characteristics of magnesium slag and achieved a calcium conversion of 30.3% for samples treated with continuous hydration under optimum process parameters. In addition, magnesium slag can be used as a raw material for the preparation of phosphate adsorbents [32], with a maximum adsorption capacity of up to 50.14 mg/g. The Fe_2_O_3_ content in magnesium slag has a large effect on the phosphorus removal rate, which can also be enhanced after acid treatment [33]. Whilst the desulfurization performance of the original magnesium slag is poor, a calcium conversion rate of up to 73.7% can be reached after mixing with additives or modification [34]. Magnesium slag, like other solid wastes, can also be used for mine filling [5,35,36] or road base material [37,38,39,40,41,42]. Numerous studies have conducted extensive research into other industrial solid wastes, such as granulated blast furnace slag [43,44,45], steel slag [46], red mud [47] and so on [48,49,50], which provide guidance for the utilization of the corresponding tailings and slag. However, there are fewer corresponding studies on the structure and cementitious properties of magnesium slag, which is one of the reasons for its current low utilization. Accordingly, there is an urgent need to study the structural characteristics of magnesium slag and propose avenues to utilize it in an efficient and resourceful way.

This work aimed to study the structural characteristics and cementitious activity of magnesium slag in comparison with those of granulated blast furnace slag using X-ray diffraction (XRD), Fourier transform infrared spectroscopy (FTIR) and ^29^Si solid-state nuclear magnetic resonance (NMR). The cementitious activity of magnesium slag and granulated blast furnace slag under alkaline conditions were assessed through the dissolution of Si, Al and Mg elements in alkaline solutions. Hydration behavior of magnesium slag and granulated blast furnace slag was investigated using scanning electron microscopy and energy dispersive X-ray spectrometry (SEM-EDS), and the possibility of their replacement for cement was also discussed. The results of this study may contribute to a deeper understanding of the relationship between the microstructure and cementitious activity of magnesium slag, which will provide basic knowledge for further comprehensive utilization of magnesium slag. Additionally, this work compares the cementitious activity of magnesium slag with that of granulated blast furnace slag for the first time, which is of great reference value to the field of magnesium slag utilization.

## 2. Experimental Program

Details of the raw materials and related test parameters used in this work are shown below.

### 2.1. Raw Materials

The magnesium slag used in the experiment was provided by Dongfeng Magnesium Metal Co., Ltd. of Yulin City, Shaanxi Province, China. The 42.5 Portland cement and granulated blast furnace slag were supplied by Henan Yuanheng Environmental Protection Engineering Co., Ltd, Henan, China. The NaOH was sourced from Yili Fine Chemicals Co., Ltd., Beijing, China. The chemical compositions of magnesium slag (MS) and granulated blast furnace slag (GBFS) as determined via X-ray fluorescence spectrometry (XRF) are shown in Table 1.

### 2.2. Characterization Methods

#### 2.2.1. Particle Size

The bulk magnesium slag was first crushed by a jaw crusher and then added into a ball mill and ground at 600 rpm for 2 h to obtain magnesium slag powder. The particle size of magnesium slag powder and granulated blast furnace slag powder was tested using a laser particle size analyzer (Bettersize2000, Dandong Baxter Instrument Co., Ltd., Dandong, China), where sodium hexametaphosphate was used as a dispersant.

#### 2.2.2. X-ray Diffraction

The mineralogical components of magnesium slag were analyzed via X-ray powder diffraction (XRD) (Bruker D8 Advance Instrument, Bruker Corporation, Karlsruhe, Germany) using Cu Kα radiation (λ = 1.54056 Å) at 40 kV and 40 mA. The scanning range was 5°~90° and the scanning speed was 5°/min.

#### 2.2.3. Nuclear Magnetic Resonance

In order to obtain more accurate solid-state NMR test results, small amounts of magnetic material were removed from the magnesium slag and granulated blast furnace slag, and then they were tested using an NMR spectrometer(AVANCE III 600M, Bruker Corporation, Karlsruhe, Germany).

#### 2.2.4. Compressive Strength

Initially, 100 g of slag powder was added to a paste mixer, stirred, vibrated and finally poured into molds. After demolding, 20 × 20 × 20 mm paste specimens were obtained, and the compressive strength of specimens at different curing ages was tested using a microcomputer-controlled electro-hydraulic servo universal testing machine (WAW-2000E, Jinan Kohui Testing Equipment Co., Ltd., Jinan, China).

#### 2.2.5. Inductively Coupled Plasma-Optical Emission Spectrometer

The active element contents (Si, Al, Mg) of magnesium slag and granulated blast furnace slag dissolved in an alkaline environment, were determined using an inductively coupled plasma instrument (ICAP-7000, Thermo Fisher, Waltham, MA, USA). The specific operations were as follows: 1 g of slag powder was added to 50 mL of 1 mol/L NaOH solution, sealed and left to stand for 72 h at room temperature, after which the upper layer of clear liquid was taken following centrifugation for ICP testing.

#### 2.2.6. Scanning Electron Microscope

The above centrifuged liquid was poured out, and anhydrous ethanol was added to terminate the hydration of the solids for 48 h, after which the solids were put into a vacuum drying oven to dry sufficiently for 24 h. The microscopic morphology of the samples was observed and analyzed using an SU 8020 field emission scanning electron microscope (SU 8020, Hitachi Ltd., Tokyo, Japan).

## 3. Results

### 3.1. Mineralogical Composition of Magnesium Slag

**Figure 1:**
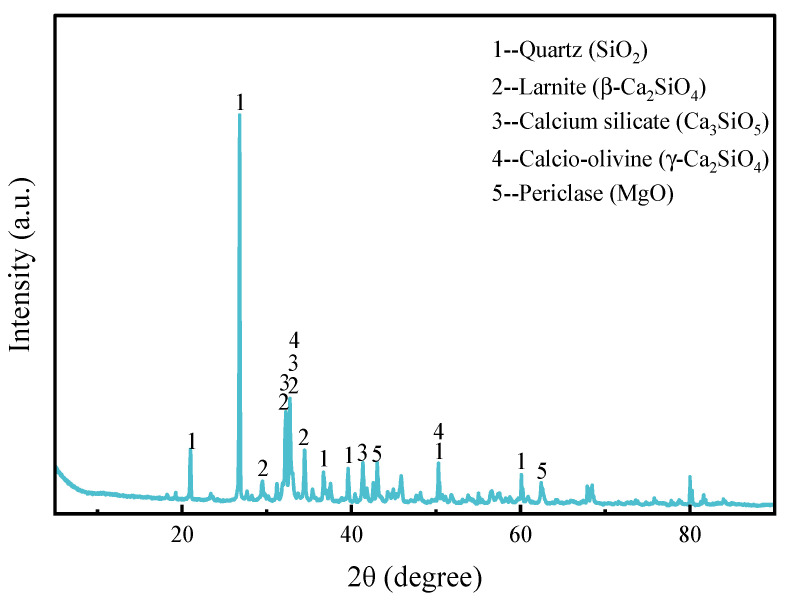
XRD pattern of magnesium slag.

Figure 1 demonstrates the XRD pattern of magnesium slag. The results show that the main phases of magnesium slag are quartz (SiO_2_), larnite (β-Ca_2_SiO_4_), calcio-olivine (γ-Ca_2_SiO_4_), calcium silicate (Ca_3_SiO_5_) and periclase (MgO). Table 2 shows the results of the quantitative XRD analysis of magnesium slag. It was found that magnesium slag contains 58.4 wt% larnite (β-Ca_2_SiO_4_), 27.1 wt% quartz (SiO_2_) and small amounts of Ca_3_SiO_5_, MgO and γ-Ca_2_SiO_4_. Compared with γ-Ca_2_SiO_4_, β-Ca_2_SiO_4_ has higher cementitious activity, and the high content of β-Ca_2_SiO_4_ in magnesium slag provides great potential for its application in cementitious materials.

**Figure 2:**
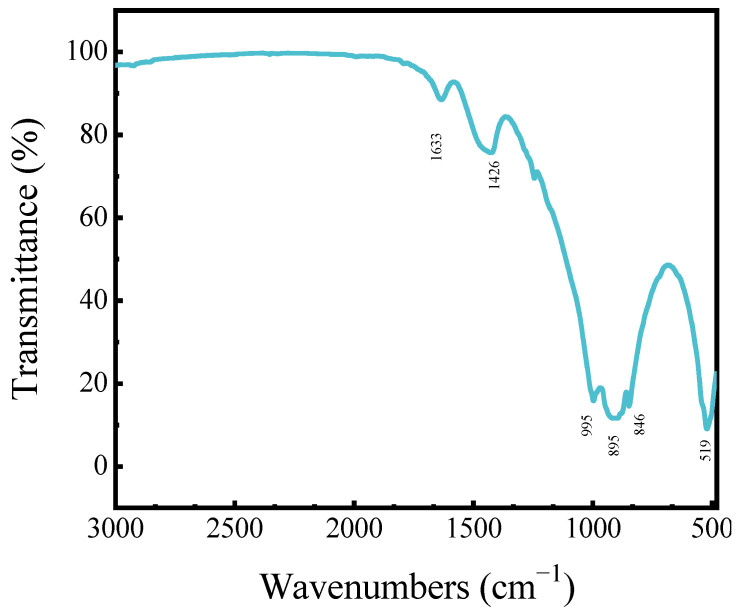
Infrared spectra of magnesium slag.

Figure 2 shows the FTIR results of magnesium slag. The main vibrational bands of magnesium slag are at 519 cm^−1^, 846 cm^−1^, 895 cm^−1^, 995 cm^−1^, 1426 cm^−1^ and 1633 cm^−1^. Among them, the Mg-O vibrational band is at 519 cm^−1^, and the stretching vibrational band of Si-O in SiO_2_ is at 995 cm^−1^. The bands at 846 cm^−1^, 895 cm^−1^ and 1426 cm^−1^ are the vibrational bands of silica–aluminum matter, and the absorption band located at 1633 cm^−1^ is related to the bending vibration of the H-O-H group of bound water.

### 3.2. [SiO_4_] Polymerization Degree of Magnesium Slag and Granulated Blast Furnace Slag

**Figure 3:**
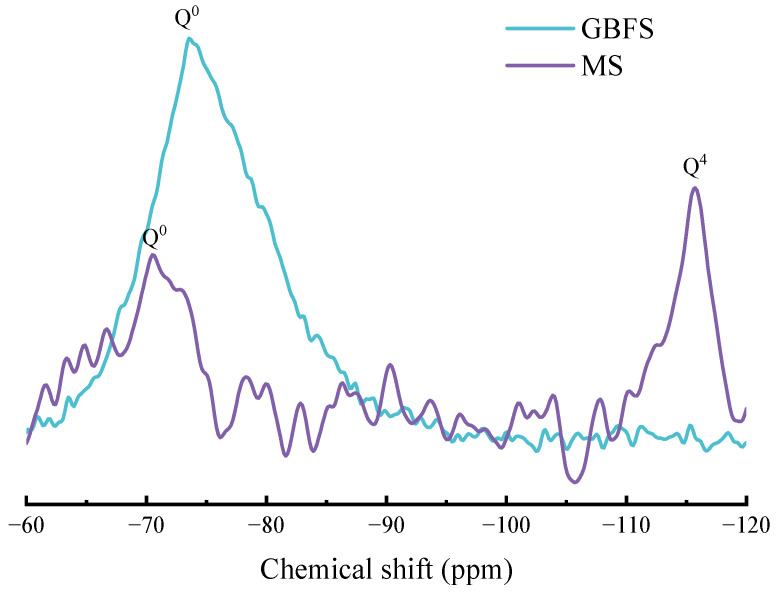
^29^Si NMR spectra of magnesium slag and granulated blast furnace slag.

In order to clarify the relationship between the cementitious activity and structure of magnesium slag, solid-state ^29^Si NMR analysis was performed to further study the [SiO_4_] polymerization degree of magnesium slag in comparison with granulated blast furnace slag [51]. Figure 3 shows the NMR spectra of magnesium slag and granulated blast furnace slag. As can be seen from Figure 3, there are two main resonance peaks in the magnesium slag. According to the relationship between their chemical shifts and structures, the resonance peak at about −70 ppm belongs to SiQ^0^, and the resonance peak at about −115 ppm belongs to SiQ^4^. The resonance peak of granulated blast furnace slag only at about −73 ppm belongs to SiQ^0^. This suggests that SiO_4_ tetrahedra in magnesium slag exist as nesosilicates and framework silicate [52], which is consistent with the quantitative analysis results of XRD.

**Figure 4:**
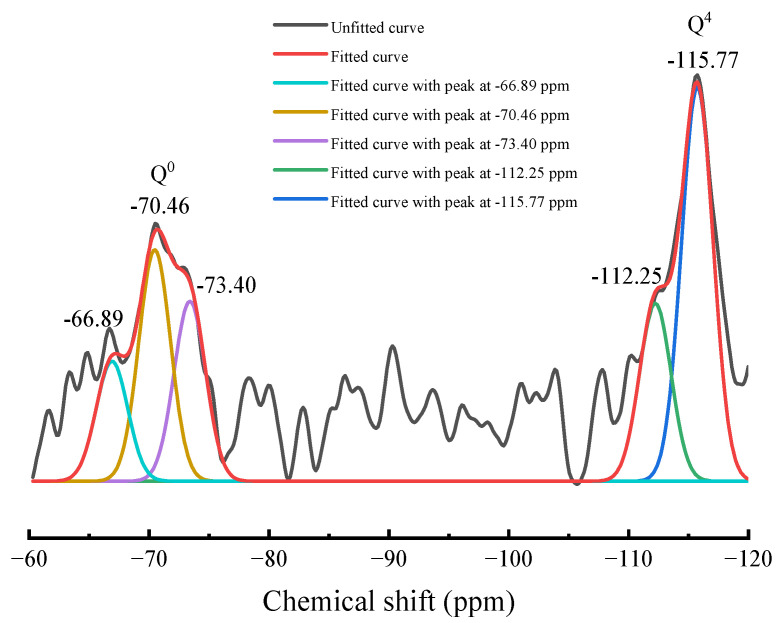
Peak-fitting results of ^29^Si NMR spectrum of magnesium slag.

Figure 4 shows the split peak fitting results of the two main peaks in the ^29^Si NMR spectrum of magnesium slag. Five independent resonance peaks were obtained by splitting the two main peaks of magnesium slag using PeakFit software (v4.04), and their areas were calculated separately. The resonance peaks at −66.89 ppm, −70.46 ppm and −73.40 ppm belong to SiQ^0^, and the resonance peaks at −112.25 ppm and −115.77 belong to SiQ^4^. The results are shown in Table 3. According to the relative bridging oxygen number (RBO) calculation formula [52],

$$RBO=\frac{1}{4}\cdot \frac{\sum n{\cdot Q}^{n}}{\sum Q^{n}}=\frac{1}{4}(1\times \frac{Q^{1}}{\sum Q^{n}}+2\times \frac{Q^{2}}{\sum Q^{n}}+3\times \frac{Q^{3}}{\sum Q^{n}}+4\times \frac{Q^{4}}{\sum Q^{n}})\;RBO(MS)=\frac{1}{4}\cdot \frac{0\cdot Q^{0}+4\cdot Q^{4}}{Q^{0}+Q^{4}}$$

it can be calculated that the RBO number of magnesium slag is 0.52. Generally speaking, the greater the relative bridging oxygen number, the higher the [SiO_4_] polymerization degree and the lower the cementitious activity of the slag. Granulated blast furnace slag is mainly composed of a SiQ^0^ unit with an RBO number of 0. Its [SiO_4_] polymerization degree is lower than that of magnesium slag, so the cementitious activity of granulated blast furnace slag is higher than that of magnesium slag.

### 3.3. Alkali-Activated Behavior of Magnesium Slag and Granulated Blast Furnace Slag

The ability of slag powder to release reactive ions ${SiO}_{4}^{4-}$ and ${AlO}_{2}^{-}$ in alkaline solution can reflect its cementitious activity [53]. In order to investigate the amount of active ions produced by magnesium slag in alkaline solution, magnesium slag powder and granulated blast furnace slag were activated with NaOH solution to assess any difference in cementitious activity. 

**Figure 5:**
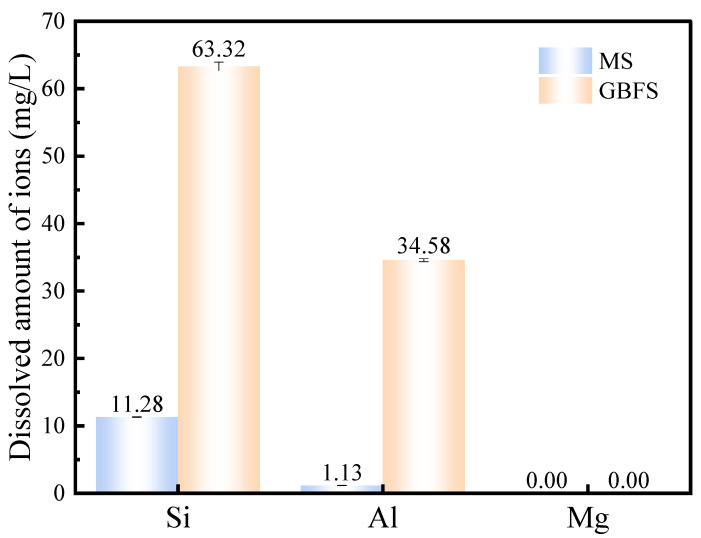
Dissolution content of Si, Al and Mg ions in 1 mol/L NaOH solution for magnesium slag and granulated blast furnace slag.

**Figure 6:**
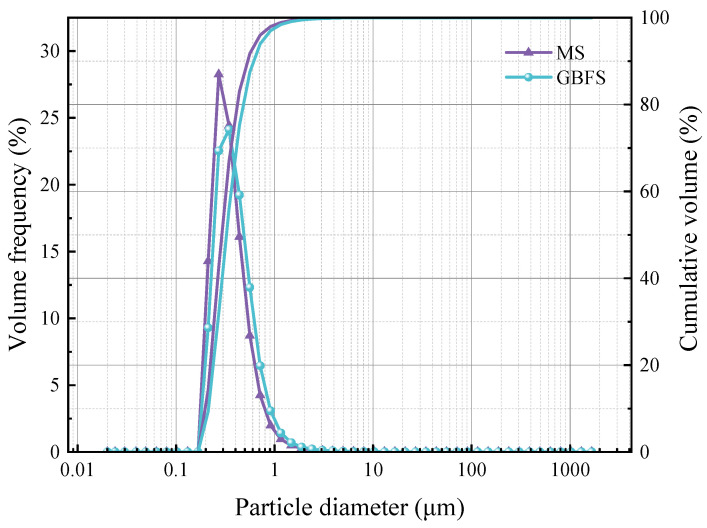
Particle size distribution of magnesium slag and granulated blast furnace slag.

The dissolution results shown in Figure 5 demonstrate both magnesium slag and granulated blast furnace slag, in which no magnesium element with cementitious activity is dissolved. Magnesium slag can dissolve a certain amount of Si and Al, while granulated blast furnace slag dissolves more. Combined with Table 1, we can surmise that the SiO_2_ content in magnesium slag is slightly higher than that in granulated blast furnace slag. However, the dissolved amount of Si in granulated blast furnace slag is about six times that of magnesium slag. As the content of Al_2_O_3_ in granulated blast furnace slag is about twenty times higher than that in magnesium slag, the dissolved amount of Al in granulated blast furnace slag is much higher than that of magnesium slag, reaching nearly 30 times more. Figure 6 shows the particle size distribution of magnesium slag and granulated blast furnace slag. It can be found that the particle size of magnesium slag powder is smaller than that of granulated blast furnace slag. In general, a smaller particle size makes it easier for a pozzolanic reaction to occur completely. However, the results of alkali dissolution are the opposite of this. These results indicate that magnesium slag powder has a certain cementitious activity, but this is much lower than that of granulated blast furnace slag.

### 3.4. Microstructure of Magnesium Slag and Granulated Blast Furnace Slag

**Figure 7:**
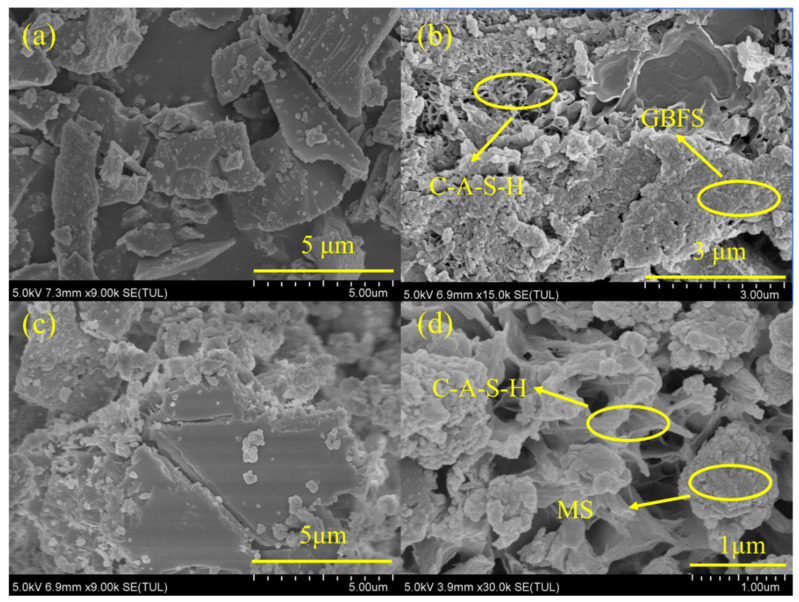
SEM images of GBFS (**a**) and GBFS after alkali dissolution (**b**); SEM images of MS (**c**) and MS after alkali dissolution (**d**).

**Figure 8:**
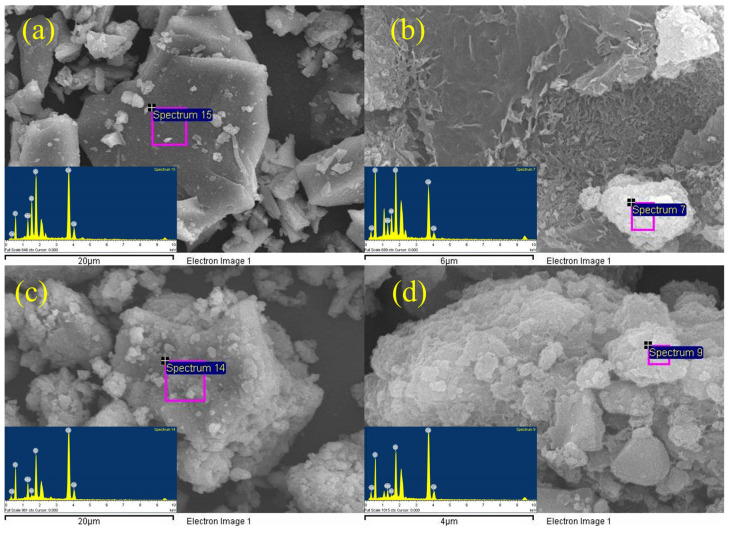
SEM-EDS analysis of GBFS (**a**) and GBFS after alkali dissolution (**b**); SEM-EDS analysis of MS (**c**) and MS after alkali dissolution (**d**).

Figure 7 shows SEM images of granulated blast furnace slag and magnesium slag samples and the corresponding samples after alkali dissolution. It can be observed that both the granulated blast furnace slag and magnesium slag were in the form of flakes with a relatively smooth surface. After alkali dissolution, the morphology of slags changed from the original flake to an agglomerate, and C-A-S-H gel formed on the surface. The slags before and after alkali dissolution were analyzed using EDS, and the results are shown in Figure 8 and Table 4. The amount of each element in the granulated blast furnace slag and magnesium slag decreased significantly after alkali dissolution, except for O, which indicates that these other elements, after alkali dissolution, are involved in the formation of C-A-S-H gel. It is noted that the consumption of each element of granulated blast furnace slag is much higher than that of magnesium slag, which may indicate that granulated blast furnace slag has higher cementitious activity than magnesium slag. This is consistent with the above ICP analysis results.

### 3.5. Magnesium Slag and Granulated Blast Furnace Slag as Replacements for Cement

**Figure 9:**
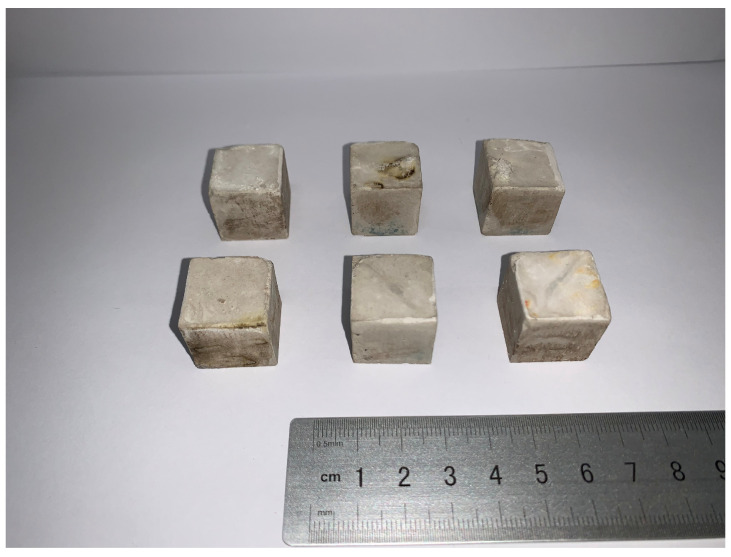
Samples for compressive strength test.

**Figure 10:**
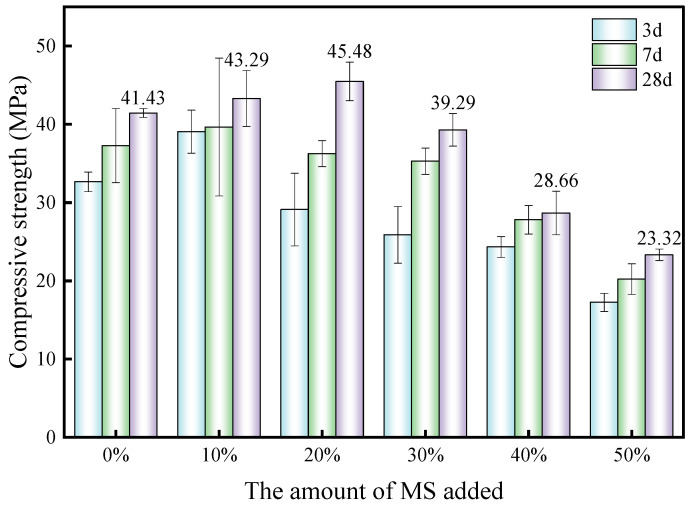
Compressive strengths of samples with different replacement amounts of magnesium slag.

**Figure 11:**
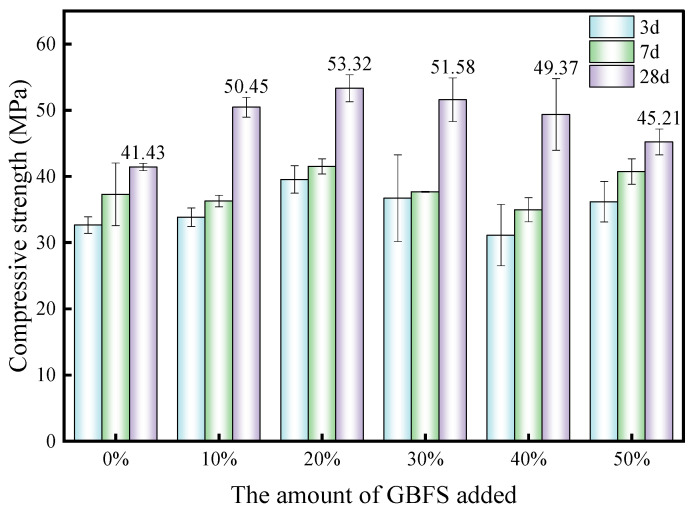
Compressive strengths of samples with different replacement amounts of granulated blast furnace slag.

In order to further confirm cementitious activity, magnesium slag and granulated blast furnace slag were used to replace a part of Portland cement in a paste experiment to compare the feasibility of their uses as a mineral admixture. Figure 9 shows samples of 20 × 20 × 20 mm paste prepared with reference to the literature [54] and standard [55], which were used for compressive strength testing after curing for the corresponding ages. Figure 10 and Figure 11 show the compressive strength of blended cement after partial replacement with magnesium slag and granulated blast furnace slag, respectively. It is beneficial to increase the compressive strength when a small amount of magnesium slag is used to replace the cement. However, the compressive strength of the samples drops below that of pure cement when more than 20% of cement is replaced by magnesium slag. The main reason is that magnesium slag has limited activity in this condition without alkali activation. When the amount of magnesium slag in the system is less than 20%, Ca(OH)_2_ produced during the hydration process of cement can play a significant role in activating the magnesium slag, so that the magnesium slag can better participate in the hydration process [56]. As a result, magnesium slag can be used to replace part of the cement within a 20% dosage whilst the compressive strength can also be improved. However, when the amount of magnesium slag continues to increase, the amount of Ca(OH)_2_ produced through cement hydration decreases, resulting in a decrease in alkali concentration in the hydrated system, and thus the activity of magnesium slag is not well-activated. Therefore, the compressive strength declines with increasing amounts of magnesium slag exceeding 20% addition. As shown in Figure 10, a similar phenomenon is observed when using granulated blast furnace slag to replace part of the cement. When the amount of replacement exceeds 20%, the 28-day compressive strength of the granulated blast furnace slag–cement samples gradually decreases with an increase in granulated blast furnace slag dosage. However, the compressive strength is still higher than that of the pure cement when a 50% dosage of granulated blast furnace slag is used for the replacement of cement, strongly indicating that granulated blast furnace slag is a valuable mineral admixture in cement and concrete. Moreover, due to the higher alkali activity of granulated blast furnace slag than that of the magnesium slag, it is found that the compressive strength of the granulated blast furnace slag–cement system is higher than that of the magnesium slag–cement group at the same replacement amount. These results suggest that although the cementitious activity of magnesium slag is lower than that of granulated blast furnace slag, magnesium slag can still be used as a mineral admixture for replacing a 20% amount of cement in blended cementitious materials. 

Various methods exist to assess the cementitious activity of solid waste, such as conductivity tests, compressive strength tests, lime absorption methods and alkali dissolution methods [57,58,59,60]. Scholars have used these methods to scientifically evaluate the cementitious activity of granulated blast furnace slag [43], steel slag [46], red mud [47] and other tailings [61], but the link between the results has not been well-identified. This work investigated the structural properties and cementitious behavior of magnesium slag and granulated blast furnace slag by using NMR, compressive strength tests and alkali dissolution methods. A difference in activity between magnesium slag and granulated blast furnace slag was demonstrated in the results. These provide a reference for magnesium slag utilization as well as assessment of the cementitious activity of other tailings.

## 4. Conclusions

This work principally investigated the structural characteristics of magnesium slag and studied its cementitious properties in comparison with granulated blast furnace slag. The main conclusions drawn are as follows:

(1) The chemical composition of magnesium slag is mainly CaO, SiO_2_, Al_2_O_3_, MgO and Fe_2_O_3_. Its main components are similar to those of granulated blast furnace slag, but it contains more MgO and less Al_2_O_3_ than granulated blast furnace slag. The main mineral compositions of magnesium slag are 27.1% quartz, 58.4% larnite, 7.1% calcium silicate, 4.8% periclase and 2.6% calcio-olivine.
(2) The Si in magnesium slag is mainly in the form of Q^0^ and Q^4^ units, which have a large relative bridging oxygen number and high [SiO_4_] polymerization degree, resulting in relatively poor cementitious activity. The Si in granulated blast furnace slag is mainly in the form of Q^0^ units, and the relative bridging oxygen number is close to 0. Compared with magnesium slag, the degree of [SiO_4_] polymerization is lower and the cementitious activity is higher for granulated blast furnace slag. After alkali excitation, the cementitious activity of magnesium slag can be reflected, and through this approach, we found that its cementitious activity is significantly lower than that of granulated blast furnace slag.
(3) Both magnesium slag and granulated blast furnace slag can be used as mineral admixtures to replace part of cement. Higher compressive strength can be obtained after replacing cement with a small amount of magnesium slag, and the optimum replacement amount is 20%. With further increase of the replacement amount, the compressive strength decreases, and when it exceeds 30%, the compressive strength of magnesium slag–cement samples is lower than that of pure cement.
(4) Magnesium slag has the potential to be utilized as a mineral admixture for cement, but attention should be paid to the amount of magnesium slag added, activity excitation and the expansion of magnesium hydrate products during its application in cement and concrete production. Elimination of the expansion effect caused by f-MgO in magnesium slag is an important topic for our future work.

## Figures and Tables

**Table 1:** Chemical composition of raw materials.

Chemical Composition (wt%)	CaO	SiO_2_	Al_2_O_3_	MgO	Fe_2_O_3_	SO_3_	TiO_2_	Others
MS	54.71	28.66	0.85	11.82	2.87	0.07	0.06	0.96
GBFS	42.76	27.85	15.61	7.78	0.36	2.64	1.20	2.16
Cement	71.52	15.08	3.59	1.81	3.44	3.03	0.43	1.10

**Table 2:** Quantitative XRD analysis of magnesium slag.

Mineralogical Composition (wt%)	SiO_2_	β-Ca_2_SiO_4_	γ-Ca_2_SiO_4_	Ca_3_SiO_5_	MgO
MS	27.1	58.4	2.6	7.1	4.8

**Table 3:** RBO calculation of magnesium slag.

Structure Units	Q^0^	Q^0^	Q^0^	Q^4^	Q^4^
Chemical shift (ppm)	−66.89	−70.46	−73.40	−112.25	−115.77
Relative area	30.42	58.74	45.64	45.14	100
Relative content (%)	10.87	20.98	16.30	16.13	35.72

**Table 4:** SEM-EDS analysis results.

Sample	O (Atomic%)	Mg (Atomic%)	Al (Atomic%)	Si (Atomic%)	Ca (Atomic%)
a	57.60	4.69	7.26	13.90	16.55
b	77.85	2.17	2.90	9.51	7.57
c	71.64	3.91	0.44	8.39	15.63
d	76.48	1.87	0.22	8.04	13.39

## Data Availability

Data are contained within the article.
